# RAP-2-independent roles for *C. elegans* MIG-15

**DOI:** 10.17912/micropub.biology.002145

**Published:** 2026-05-15

**Authors:** Razan A. Fakieh, Ranjana Kishore, Meera V. Sundaram, David J. Reiner

**Affiliations:** 1 Vashisht College of Medicine, Texas A&M University, Houston, TX USA; 2 Department of Genetics, University of Pennsylvania, Philadelphia, PA, USA; 3 Current address: Division of Biology and Biological Engineering, California Institute of Technology, Pasadena, CA 91125, USA; 4 Institute of Biosciences and Technology, Texas A&M Health Science Center, Houston, TX, USA

## Abstract

MIG-15 is the sole *Caenorhabditis elegans* member of the GCK-IV subfamily of Ste20 kinases. In mammals and *in vitro* , MIG-15-like kinases can function as effectors of the small GTPase Rap2. To test this model *in vivo* , we compared phenotypes of *mig-15* and *rap-2* mutants. *mig-15* mutants displayed severe defects in vulval morphogenesis, cell positioning, and locomotion. In contrast, *rap-2* mutants were largely indistinguishable from wild type. These findings indicate that several developmental roles of MIG-15 occur independently of RAP-2, suggesting that additional upstream regulators, including other small GTPases or adhesion-related pathways, control MIG-15 activity in specific developmental contexts.

**Figure 1. mig-15 mutants reveal vulval and locomotion defects not observed in rap-2 mutants:** **(A-C)** Representative DIC photomicrographs of P6.px cells and the AC in mid-L3 animals used for assessing centering of the AC and the presumptive 1˚ lineage. Arrowheads indicate anchor cells (ACs) and brackets indicate P6.p daughters P6.pa and P6.px. **(A)** Wild type, **(B)** *mig-15(rh148)* , and **(C)** *rap-2(re400)* . **(D-F)** Schematics of P6.px centering on the AC for the same genotypes as **A** , **B** , **C** , respectively. **(G-I)** Representative DIC photomicrographs of vulval morphogenesis at the mid-L4 stage for the same genotypes as **A** , **B** , **C** , respectively. **(J)** Quantification of displacement of the midline between P6.pa and P6.pp from the midline of the AC reveals a strong defect in the *mig-15* but not *rap-2* mutants shown in **A-F** . **(K)** A radial locomotion assay of *rap-2* vs. *mig-15* mutants revealed severe locomotion defects in *mig-15* mutants but none in *rap-2* mutants. Distance shown is in mm from the origin at which animals were placed vs. final position after 20 minutes on 10 cm plates. N = 30 for each genotype. **(L)** DIC analysis of the wild type vs. *rap-2* and *mig-15* mutants for Vulvaless (Vul) and Abnormal (Abn) phenotypes (see Methods). ^‡^ These data were also presented in Fakieh and Reiner, 2025. ^∞^ *rhIs15* is a MIG-15::GFP over-expressing (OE) integrated transgene from Xiaoping Zhu and Edward Hedgecock (Zhu, 1998). ^§^ A small percentage (< 20%) of these animals showed multiple invaginations. ^Φ^ 76% of these animals showed multiple invaginations. **(M)** Cell lineage analysis of P5.px, P6.px and P7.px cell divisions in vulval development during the late L3 stage. *mig-15* mutants consistently show defects in the axes of this final cell division. The nomenclature used to describe the plane of cell divisions is L= longitudinal, T = transverse, O = oblique, N = no division. ANOVA was used for statistical analysis. **** represents p ≤ 0.0001, * represents p ≤ 0.05.

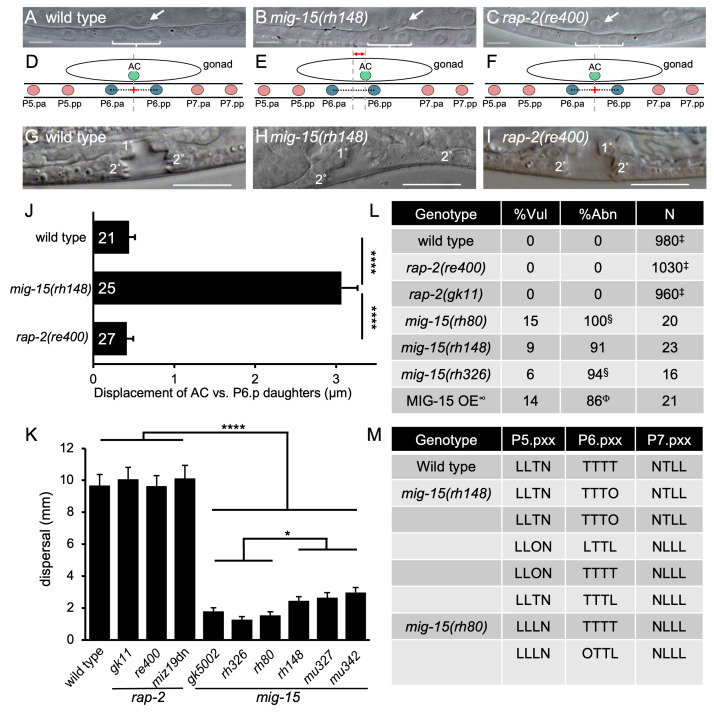



## Description

MIG-15 is the sole *Caenorhabditis elegans* representative of the GCK-IV subfamily of Ste20 S/T kinases, which is conserved across metazoans. These proteins consist of an N-terminal Ste20 kinase domain, a long central proline-rich linker, and a C-terminal CNH domain (Citron-NIK Homology) &nbsp;(Chuang et al., 2016; Dan et al., 2001; Delpire, 2009). The *Drosophila* ortholog is Misshapen (Msn) and the mammalian orthologs are MAP4K4 (HGK/NIK), MAP4K6 (MINK1), and MAP4K7 (TNIK) (Bunardi et al., 2025). (NRK/NESK, sometimes referred to as MAP4K8, is generally not included in this group because its sequence is more divergent and expression is enriched in placenta (Denda et al., 2011), whereas the other GCK-IV kinases are broadly expressed.) The paralogous GCK-I subfamily of Ste20 kinases, consisting of *C. elegans* GCK-2 , *Drosophila* Happyhour (Hppy) and mammalian MAP4K1,2,3,5, is structurally similar but functionally distinct.

MIG-15 in *C. elegans* regulates diverse morphogenetic and developmental processes (Chapman et al., 2008; Crawley et al., 2017; DaCunha et al., 2025; Huynh et al., 2026; Poinat et al., 2002; Shakir et al., 2006; Teuliere et al., 2011; Yang et al., 2014). Similar roles have been defined for *Drosophila* Msn (Kline et al., 2018; Paricio et al., 1999; Ruan et al., 2002; Su et al., 2000; Su et al., 1998).

The small GTPase Rap2 has been shown in mammalian systems and *in vitro* to bind or be phenotypically associated with MIG-15-like proteins of the GCK-IV subfamily. These findings indicate that, in mammals, GCK-IV Ste20 kinases can function as effectors of Rap2 (Gloerich et al., 2012; Hussain et al., 2010; Machida et al., 2004; Meng et al., 2018; Nonaka et al., 2008; Pannekoek et al., 2013; Taira et al., 2004). Recent studies make phenotypic connections between *Drosophila* Rap2l and Msn (Roberto et al., 2025), as well as between *C. elegans* RAP-2 and MIG-15 in synaptic tiling (Chen et al., 2018) and cell fate induction (Fakieh and Reiner, 2025).

During our studies, we observed that *mig-15* mutant animals exhibit morphogenetic defects – in body shape, locomotion, and vulva – while *rap-2* mutant animals are superficially wild type. Such a discrepancy is unexpected for a small GTPase and its effector, which often share loss-of-function phenotypes. Consequently, we characterized phenotypic defects in *mig-15* relative to *rap-2* mutant animals. These studies used putative *mig-15* null mutations ( *gk5002* , *rh80 ,* *rh326* ) and missense mutations in conserved residues in the kinase domain ( *rh148* , *mu327* , *mu342* ), and putative *rap-2* null mutations ( *gk11 ,* *re400* ) and a dominant-negative allele ( *miz19* ) (see Methods). Results were consistent across the multiple alleles tested.

Six ventral vulval precursor cells (VPCs), P3.p through P8.p, are spaced along the ventral midline during early larval development. Signal from the Anchor Cell (AC) induces these VPCs to assume the 3˚-3˚-2˚-1˚-2˚-3˚ pattern of VPC fates with 99.8% accuracy (Braendle and Felix, 2008; Shin et al., 2019). P6.p, closest to the AC, typically assumes the 1˚ fate, while the neighboring P5.p and P7.p cells assume the 2˚ fate (Shin and Reiner, 2018). During the L2 and early L3 stages, prior to its induction to assume the 1˚ fate, P6.p migrates to be positioned ventral to the AC (Grimbert et al., 2016). When assayed after the first VPC division, the P6.p daughters P6.pa and P6.pp were frequently displaced in *mig-15 ( rh148 )* animals. In contrast, the P6.p daughters P6.pa and P6.pp in *rap-2 ( re400 )* animals were positioned with the same accuracy as in wild type ( **Figure 1 A-C** , schematized in **Figure 1D- F** and quantified in **Figure 1J** ). The mid-L4 invaginated vulva, prior to eversion, forms a stereotyped structure that is frequently defective in *mig-15 ( rh148 )* and other *mig-15* mutants, but not a *rap-2* mutant ( **Figure 1G- I** ; see also (Shin et al., 2018), quantified in **Figure 1L** . These defects included missing vulva cells (Vul phenotype) and, more frequently, mis-positioned vulva cells (Abn phenotype, **Figure 1H** ). Overexpression of *mig-15* (with transgene *rhIs15* , see Methods) caused similar vulva defects ( **Figure 1L ).** Analysis of VPC cell lineages by examining planes of cell division at the Pn.pxx cell division revealed defects in *mig-15* mutants ( **Figure 1M** ).

By visual inspection, *mig-15* mutant animals move poorly, while *rap-2* mutants move normally. To assess general nervous system function via locomotion (Mardick et al., 2021), we measured radial locomotion of animals placed at the center of a plate. *rap-2* mutants moved normally, while *mig-15* mutants exhibited severe locomotion defects ( **Figure 1K** ). The putative null *mig-15* mutations ( *gk5002,* *rh80 ,* *rh326* ) confer marginally more severe locomotion defects than the missense mutations ( *rh148* , *mu327* , *mu342* ).

Given the established relationships between Rap2 and MIG-15 /GCK-IV orthologs, it is striking to observe loss-of-function *mig-15* mutant phenotypes not phenocopied by loss-of-function mutations in *rap-2 .* Consequently, we entertain the possibility that RAP-2 activates MIG-15 in only a subset of MIG-15 functions. Alternatively, RAP-2 could function redundantly with other inputs to control MIG-15 in selected tissues. MIG-15 mutant morphogenetic defects resemble those caused by mutations in MIG-2 /RhoG and CED-10 /Rac, as well as their activating RhoGEF UNC-73 /TRIO (Kishore and Sundaram, 2002). This phenotypic similarity hints that these Rho family GTPases could activate MIG-15 in particular developmental contexts, in contrast to the Ras family GTPase Rap2.

By yeast two-hybrid assay, the CNH domain of MIG-15 interacts with the cytoplasmic domains of INA-1 ⍺ and PAT-3 β integrin subunits, predicted to form a laminin-binding integrin (Poinat et al., 2002). These interactions were supported by *in vitro* assays and experiments in HeLa and COS cells (Poinat et al., 2002). Genetic interactions in *C. elegans* are consistent with MIG-15 and INA-1 / PAT-3 acting in the same pathway in axon guidance and fasciculation.

Mammalian TRAF1 (TNF receptor associated factor; (Inoue et al., 2000)) binds TNIK/MAP4K7 as an activator during inflammatory response (Fu et al., 1999), suggesting *C. elegans* TRAF orthologs TRF-1 and/or TRF-2 (Nikonorova et al., 2025) as potential upstream inputs. A negative regulator of GCK-IV MIG-15 subfamily proteins is the SH2-SH3 domain adaptor protein NCK, which binds to the central proline-rich region to sequester the kinase (Su et al., 1997). Release from this inhibition could activate MIG-15 in selected tissues.

Our results demonstrate that MIG-15 controls multiple morphogenetic and developmental processes in *C. elegans* that are not detectably dependent on RAP-2 . These findings suggest that additional upstream regulators contribute to MIG-15 function *in vivo* , as well as in other systems where MIG-15-like GCK-IV subfamily proteins perform important functions.

## Methods

**Animal handling.** All strains were derived from the N2 Bristol wild type. Animals were grown at 20 ˚C on OP50 *E. coli* seeded on NGM plates. Nomenclature was as described (Tuli, Daul, & Schedl, 2018). Wormbase and the Alliance of Genome Resources were both used (Sternberg et al., 2024).

The *rap-2 ( gk11 )* knockout consortium deletion was backcrossed 5x to the wild type, *rap-2 ( re400 )* is a STOP-IN disruption generated via CRISPR and *rap-2 ( miz19* dn *)* is a dominant negative mutation generated by CRISPR (Chen et al., 2018; Fakieh & Reiner, 2025). *mig-15 ( rh148 )* , *mig-15 ( mu327 )* and *mig-15 ( mu342 )* are kinase domain missense alleles, *mig-15 (gk5002)* is a gene replacement, and *mig-15 ( rh326 )* and *mig-15 ( rh80 )* are nonsense alleles, all published previously (Fakieh & Reiner, 2025).

**Imaging.** Mid-L3, and mid-L4 animals were mounted on 3% agar pads containing 10 mM sodium azide as described (Sulston & Horvitz, 1977). Animals were scored using Differential Interference Contrast (DIC)/Nomarski optics on a Nikon Eclipse Ni microscope. Images were captured with an Andor Zyla camera and analyzed using Nikon NIS-Elements AR 4.20.00 software.

**AC-VPC Centering assay.** Animals were imaged using DIC microscopy at the Pn.px stage. Using the Nikon NIS Elements Advanced Research software, we measured the distance in microns between center of the AC nucleus and the mid-point between nuclei of the P6.pa and P6.pp daughter cells of P6.p.

**VPC lineaging:** The cell division planes of P5.pxx, P6.pxx and P7.pxx were observed by DIC/Nomarski microscopy. Nomenclature used to describe the polarity of cell divisions was L = longitudinal, T = transverse, O = oblique, and N = no division (Sternberg & Horvitz, 1986). By the L4 stage, vulval cell divisions and the invagination are normally complete. The numbers of vulval and non-vulval VPC descendants were counted to assess defects in cell-fate specification. Identities of individual nuclei were inferred based on their position and morphology and axes of final vulval divisions were determined by direct observation *.*

**Radial locomotion assay.** Locomotion was assayed as described (Mardick et al., 2021; Reiner, Newton, Tian, & Thomas, 1999; Reiner et al., 2006). Briefly, hermaphrodite adults without eggs were placed in the center of a 10-cm plate with a three-day evenly distributed lawn of OP50 *E. coli* and the origin was marked. Animals were allowed to move freely on the plate for 20 min at 20 ˚C. The plates were then transferred to -20 ˚C for 5 min to arrest movement. The final location of each animal was marked and the radial distance from the origin to the final point was measured to the nearest half mm. Statistical analysis was performed using Mann-Whitney U test and ANOVA (see figure legends for P values).

## Reagents

**Strains used**

All strains but NJ824 used for this study are described in (Fakieh & Reiner, 2025).

DV4144 &nbsp;&nbsp;&nbsp;&nbsp;&nbsp;&nbsp;&nbsp;&nbsp;&nbsp; *rap-2 ( re400 )*

DV3054 &nbsp;&nbsp;&nbsp;&nbsp;&nbsp;&nbsp;&nbsp;&nbsp;&nbsp; *rap-2 ( gk11 )* (5x backcrossed to N2 )

UJ402 &nbsp;&nbsp;&nbsp;&nbsp;&nbsp;&nbsp;&nbsp;&nbsp;&nbsp;&nbsp;&nbsp;&nbsp; *rap-2 ( miz19* dn *)*

DV3999 &nbsp;&nbsp;&nbsp;&nbsp;&nbsp;&nbsp;&nbsp;&nbsp;&nbsp;&nbsp; *mig-15 (gk5002 [gkIs267( mig-15 :: myo-2* p *>GFP:: mig-15 )])* (first-to-last-exon replacement)

NJ834 &nbsp;&nbsp;&nbsp;&nbsp;&nbsp;&nbsp;&nbsp;&nbsp;&nbsp;&nbsp;&nbsp;&nbsp; *mig-15 ( rh326 )*

NJ298 &nbsp;&nbsp;&nbsp;&nbsp;&nbsp;&nbsp;&nbsp;&nbsp;&nbsp;&nbsp;&nbsp;&nbsp; *mig-15 ( rh80 )*

NJ490 &nbsp;&nbsp;&nbsp;&nbsp;&nbsp;&nbsp;&nbsp;&nbsp;&nbsp;&nbsp;&nbsp; *mig-15 ( rh148 )*

CF1665 &nbsp;&nbsp;&nbsp;&nbsp;&nbsp;&nbsp;&nbsp;&nbsp;&nbsp;&nbsp; *mig-15 ( mu327 )*

CF1667 &nbsp;&nbsp;&nbsp;&nbsp;&nbsp;&nbsp;&nbsp;&nbsp;&nbsp;&nbsp; *mig-15 ( mu342 )*

NJ824 &nbsp;&nbsp;&nbsp;&nbsp;&nbsp;&nbsp;&nbsp;&nbsp;&nbsp;&nbsp;&nbsp;&nbsp; *rhIs15 [ mig-15 ::GFP* OE *]*
